# An Exploratory Study on Changes in Patients in the Emergency Department due to Coronavirus Disease 2019 in Japan: A Single-center Retrospective Study

**DOI:** 10.31662/jmaj.2022-0075

**Published:** 2022-09-26

**Authors:** Michihiro Tsubaki, Yu Haniuda, Ai Ushiwata, Takuya Mori, Ryoko Ikari, Rumi Tanaka, Satoshi Tamura

**Affiliations:** 1 School of Nursing, Kitasato University, Sagamihara, Japan; 2 Department of Emergency Nursing, Kitasato University Hospital, Sagamihara, Japan; 3 School of Pharmacy, Kitasato University, Tokyo, Japan; 4 Department of Emergency and Critical Care Medicine, Kitasato University School of Medicine, Sagamihara, Japan

**Keywords:** Emergency, Hospital visits, COVID-19

## Abstract

**Introduction:**

This study assessed changes in patients transported to an emergency and critical care center before and after the coronavirus disease 2019 (COVID-19) pandemic in Japan and examined problems that should be addressed in emergency medical care.

**Methods:**

This single-center retrospective observational study was conducted at a university hospital. The subjects were patients who were transported to a “tertiary emergency department” receiving advanced medical care. With January 16, 2020, as the cutoff date, 4,197 patients who were transported to the hospital from January 16, 2019, to January 15, 2021, were recruited. The patients were classified into nine disease groups using the International Classification of Diseases, Tenth Revision. The emergency department (ED) visit count in 2020 was compared with that in 2019 using Poisson regression.

**Results:**

The number of patients transported to the tertiary ED in 2020 decreased by 7.8% compared with that in 2019. The number of patients transported to the tertiary ED decreased compared with that in the previous year during the period when the number of confirmed COVID-19 cases in Japan increased and showed the opposite trend when the number of confirmed cases decreased. As per diagnostic classification, it decreased for all diagnoses except External causes, and significant decreases were observed in Infectious (47.5%), Non-COVID-19 respiratory (28.4%), and Trauma (25.2%). In External causes, the rate of change for suicide cases alone increased to 43.2%.

**Conclusions:**

While the number of confirmed cases increased, the number of tertiary ED patients associated with COVID-19 decreased temporarily. It is necessary to fully consider the burden on medical institutions 1-2 months after the number of infected people peaks. It is also necessary to closely monitor suicides associated with COVID-19 as a factor that will cause changes in emergency medical care in the future.

## Introduction

Coronavirus disease 2019 (COVID-19) has triggered a pandemic, and the transmission of the infection could not be controlled for a long time ^(1)^. This pandemic, which will be widely remembered in human history, has to a large extent affected healthcare and economies ^(2)^. Above all, the increased hospital bed occupancy rate in intensive care units owing to the sharp increase in the number of critically ill patients has become a massive problem ^(3)^. COVID-19 has resulted in the collapse of medical resources and lockdowns worldwide ^(4)^ owing to its extremely rapid transmission and high rate of short-term mortality ^(1)^. In addition to the impact *per se* of the disease, which is the mere effect of a virus, there is a psychological impact, which results from the fact that the virus is not visible and that the treatment method has not yet been established, accompanied by a social impact caused by hatred, discrimination, and bias resulting from anxiety and fear about COVID-19, as well as infection control ^(5), (6)^. These effects have altered human behavior, and these changes have also extended to the cases of patients being transported to the emergency department (ED) at hospitals. In areas affected by the rapid transmission of COVID-19, with the increase in the number of confirmed COVID-19 cases, the number of patients transported to emergency and critical care centers has reportedly decreased ^(7), (8), (9)^. Thus, although changes in the number of patients transported to the ED caused by the transmission of COVID-19 have been verified worldwide, no such investigation has been conducted in Japan. Considering that the status of COVID-19 differs widely among countries and different governments have diverse policies, verifications tailored to the situation of each country would be useful.

In addition, establishing a suitable medical system is an important problem that needs to be addressed amid the COVID-19 outbreak ^(10)^. In particular, the verification of patient acceptance capacity associated with the medical system is an important strategy ^(11)^, and multidimensional verifications are essential. Patients with COVID-19 in Japan are characterized by low severity and mortality rate compared with those in other countries ^(12)^. Nevertheless, a shortage of medical institutions and medical personnel occurred even in emergency medical care settings, and cases in which medical conditions changed unexpectedly during home recovery have been reported ^(13)^. Investigating the effect of COVID-19 on emergency medical care would enable adapting to the situation of emergency patients, which has changed because of COVID-19. In particular, the examination of appropriate operation strategies of emergency and critical care centers, which is a limited medical resource, is an important problem that should be addressed to maintain medical systems in the future. Thus, this study aimed to clarify the changes in the number of patients transported to an emergency and critical care center before and after the COVID-19 pandemic in Japan and investigated problems that need to be addressed in emergency medical care in the future.

## Materials and Method

### Study design and setting

This study was a single-center retrospective observational study conducted at a university hospital with an emergency and critical care center in the Tokyo Metropolitan Area, Japan. The hospital that conducted the survey is a large university hospital with more than 1,000 beds. The institution records approximately 2,000 tertiary ED visits annually that require advanced medical care and is responsible for the majority of advanced critical care in the surrounding medical area. Because there is no other tertiary emergency center in the city, tertiary emergency care is rarely refused even if beds are full. As a result, it is less likely that the transported patient will change the medical institution for convenience. It is located in a major city in Japan and is thus positioned at the epicenter of disease transmission. Prior to the study initiation, an opt-out of the study was posted on the hospital’s website to ensure that the enrolled subjects had the opportunity to refuse participation in the study. This retrospective study was approved by the Institutional Review Board for Observation and Epidemiological Study, Kitasato University Medical Ethics Organization (B20-326).

### Epidemiological scenario and lockdown timing in Japan

**Figure 1.**
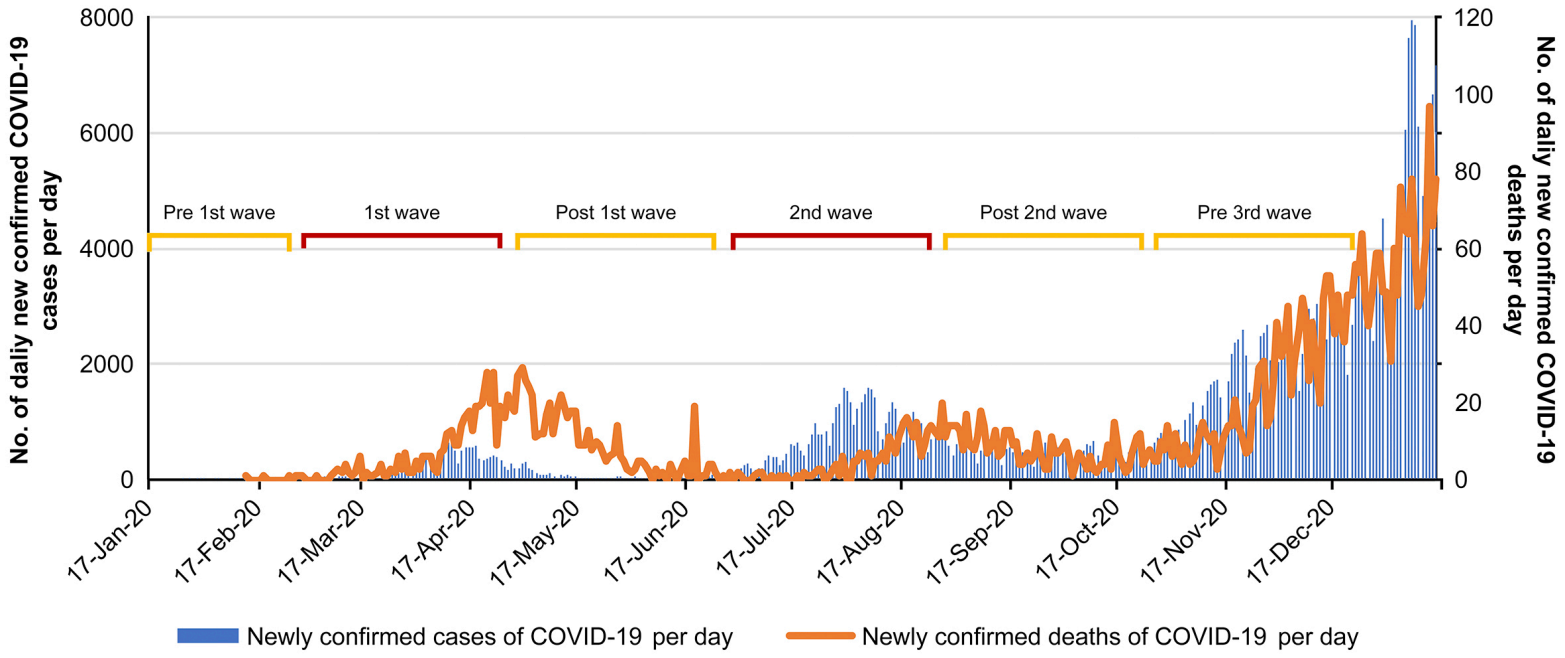
Epidemiological scenario of COVID-19 pandemic in the capital region of Japan from January 2020 to February 2021.

The number of confirmed cases in Japan had increased sharply since late March 2020, when the first wave of the COVID-19 pandemic hit the nation. On April 7, 2020, the Japanese government declared a state of emergency in major cities. Subsequently, on April 16, 2020, it was expanded to the entire nation. On May 25, 2020, the state of emergency was withdrawn nationwide. Although the number of confirmed cases in Japan was maintained at a level lower than that in other countries, a second state of emergency was declared on January 8, 2021 ^(14)^. Figure 1 shows the number of confirmed cases and the number of confirmed deaths in the capital of Japan ^(15)^.

COVID-19 also had a significant impact on the facilities surveyed. Emergency medical staff wore infection protection equipment for all responses and performed rapid antigen tests on all emergency transport patients. Patients requiring hospitalization waited in the ED until their COVID-19 report was confirmed to be negative. Cases suspected of being infected due to fever or contact history were treated in a private room with independent ventilation. All emergency services used by the patients were managed in private rooms and disinfected after each use. The number of patients with COVID-19 accepted by the target facility during the survey period was 289. In this study, the phases of infection in Japan were classified into five stages. January to February, before the spread of the infection, was defined as the pre-first wave; March to April, when the initial spread of the infection occurred, was defined as the first wave; May to June, when the spread of the infection was converging, was defined as the post-first wave; July to August, when the spread of infection occurred the second time, was defined as the second wave; September to October, when the second wave was converging, was defined as the post-second wave, and November to December, the period of the spread of infection before the third wave, was defined as the pre-third wave.

### Measurements

The study period was 2 years, i.e., from January 16, 2019, to January 15, 2021, with January 16, 2020, when the infection was first confirmed in Japan, regarded as the standard. In this study, the period of 365 days following January 16, 2020, which was impacted by COVID-19, was expressed as 2020, and the period of 365 days before the first confirmation of COVID-19 infection in Japan was expressed as 2019. Emergency transport data were extracted from Fortec ACSYS, the intensive care unit patient information system, in an anonymized format at the target institution. The extracted items were sex, age, disease name at admission, and date and time of hospital arrival. Patients positive for COVID-19 were excluded from the study because they differed from the conventional emergency transport route.

### Diagnostic classification

Diagnostic classification was performed using the International Classification of Diseases, Tenth Revision (ICD-10) with reference to a previous study ^(16)^, and very few changes were made based on clinical reasoning. From 22 major categories, 7 with a high frequency of transport to emergency and critical care centers were extracted. In addition, “Poisoning by drugs, medicaments, and biological substances (Poisoning by drugs),” which has a high transport frequency, and “others,” which do not correspond to any of the above, were added. The specific categories are shown in Table 1 Stroke, cerebrovascular diseases, and cardiopulmonary arrest on arrival at the hospital, excluding those with a clear cause, were classified as “Diseases of the circulatory system” (I00-I99: Cardiovascular). Among cases of cardiopulmonary arrest on arrival at the hospital, those caused by self-harm or trauma were classified as “External causes of morbidity and mortality” (V01-Y98: External causes).

**Table 1.** Diagnostic Classification of the International Classification of Diseases, Tenth Revision.

Code	Diseases	Abbreviation
A00-B99	Certain infectious and parasitic diseases	Infectious
G00-G99	Diseases of the nervous system	Neurological
I00-I99	Diseases of the circulatory system	Cardiovascular
J00-J99	Diseases of the respiratory system	Non-COVID-19 respiratory
K00-K93	Diseases of the digestive system	Gastrointestinal
S00-T98	Injury, Poisoning, and certain other consequences of external causes	Trauma
V01-Y98	External causes of morbidity and mortality	External causes
Not applicable	Poisoning by drugs, medicaments, and biological substances	Poisoning by drugs

### Statistical analysis

The ED visit counts in 2020 and 2019 were compared using Poisson regression, and the daily ED visit counts were considered the dependent variable and the year, the independent variable. As 2020 was a leap year, data on February 29 were excluded to unify the target periods. In Poisson regression, the estimated value of the regression coefficient corresponds to the logarithm of the ratio of ratios. Therefore, the percentage change in 2020 relative to 2019 and its 95% confidence interval was derived from this estimate. Type-III p-value was used to assess the presence of statistically significant changes (p < 0.05).

## Results

### Visit number and characteristics

**Figure 2.**
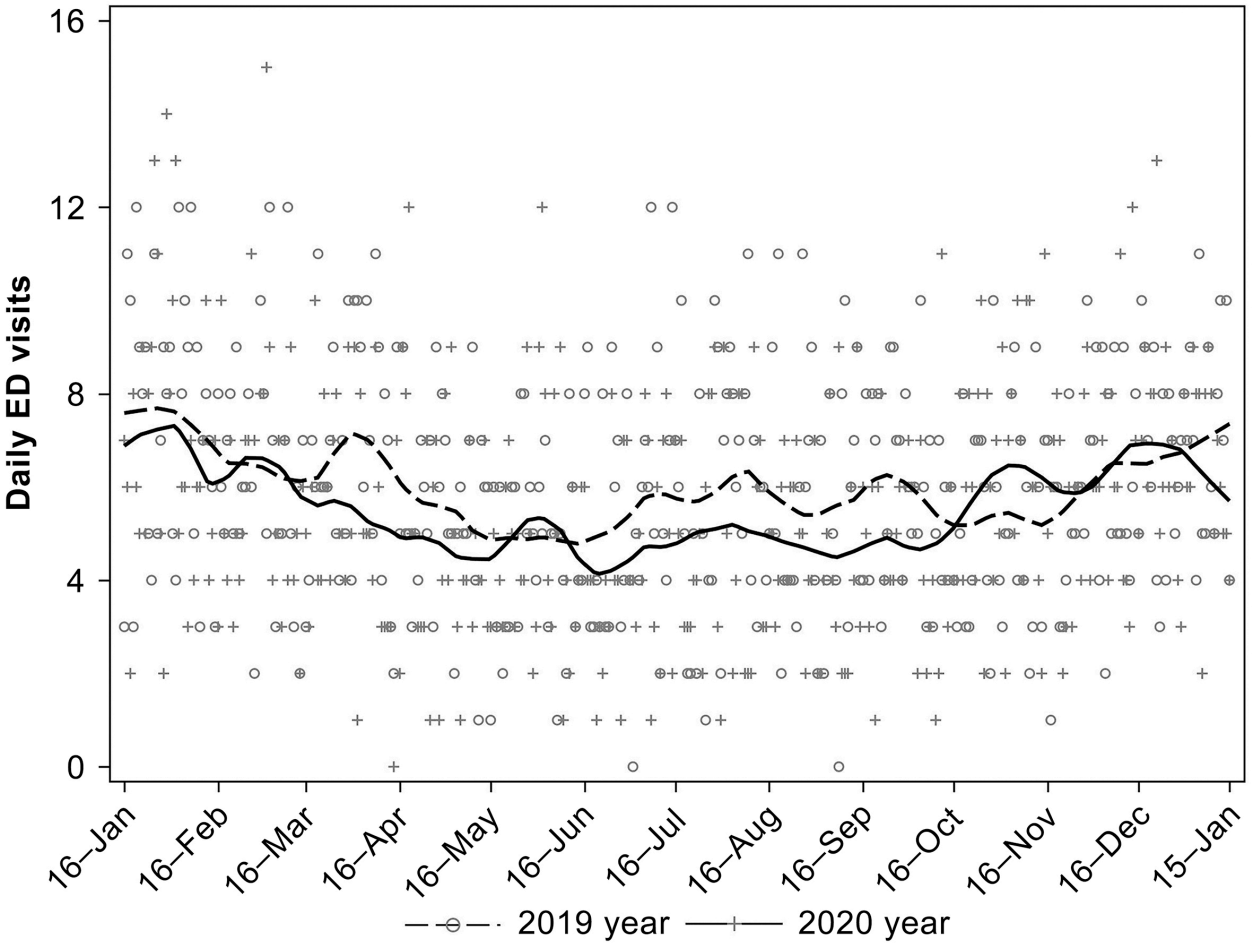
Daily number of emergency department (ED) visits in 2020 and 2019.

The number of patients transported to the tertiary ED within the target period was 2,013 in 2020 and 2,184 in 2019. The number of patients transported to the tertiary ED throughout 2020 decreased by 7.8% compared with that in 2019 (p = 0.013; Table 2). The number of patients transported to the tertiary ED had reduced compared with that in the previous year during the periods when the first and second waves occurred and increased compared with that in the previous year at the post-first and post-second waves (Figure 2).

**Table 2.** Number of Emergency Department (ED) Visits Classified by Final Diagnosis in 2020, with Corresponding Percent Changes in 2019.

ED final diagnosis	Number of ED visits in 2019	Number of ED visits in 2020	Percent change	95% confidence interval		P-value
				Lower	Upper	
Total number of patients	2,184	2,013	−7.8%	−13.5	−1.7	0.013
Infectious	59	31	−47.5%	−65.3	−20.4	0.002
Neurological	20	15	−25.0%	−61.0	44.3	0.386
Cardiovascular	986	949	−3.8%	−12.5	5.9	0.430
Non-COVID-19 respiratory	102	73	−28.4%	−47.2	-2.9	0.030
Gastrointestinal	128	106	−17.2%	−36.0	7.1	0.150
Trauma	159	119	−25.2%	−41.1	−4.9	0.017
External causes	368	389	5.7%	−8.2	21.7	0.441
Poisoning by drugs	162	160	−1.2%	−21.1	23.7	0.914
Others	200	171	−14.5%	−30.3	4.8	0.131

※Excluding data as of February 29, 2020

### Monthly visits

**Figure 3.**
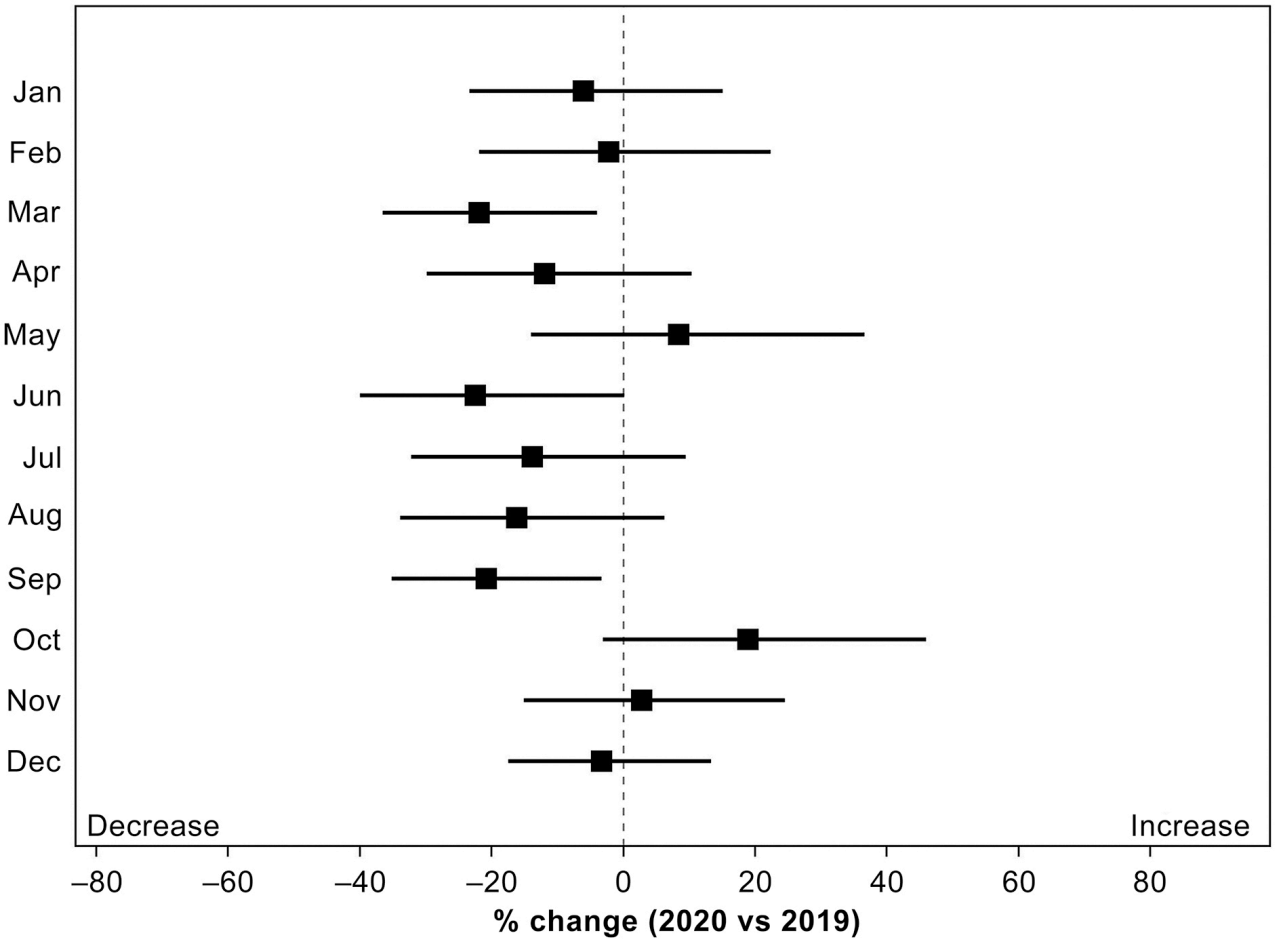
Percent change (with 95% confidence interval) in emergency department (ED) transport patients other than those with COVID-19.

The rate of change in the number of patients transported to the tertiary ED by month fluctuated according to the number of confirmed COVID-19 cases, which was similar to the results of locally estimated scatterplot smoothing (LOESS) (Figure 3). The number of patients transported to the tertiary ED significantly decreased in March, which corresponded to the first wave, and in September, which corresponded to the second wave. As mentioned above, even though the number of patients transported to the tertiary ED throughout the year decreased, the number of patients transported to it increased compared with that in the previous year (2019) in May, October, and November, when the number of confirmed COVID-19 cases decreased.

### ED diagnoses

**Figure 4.**
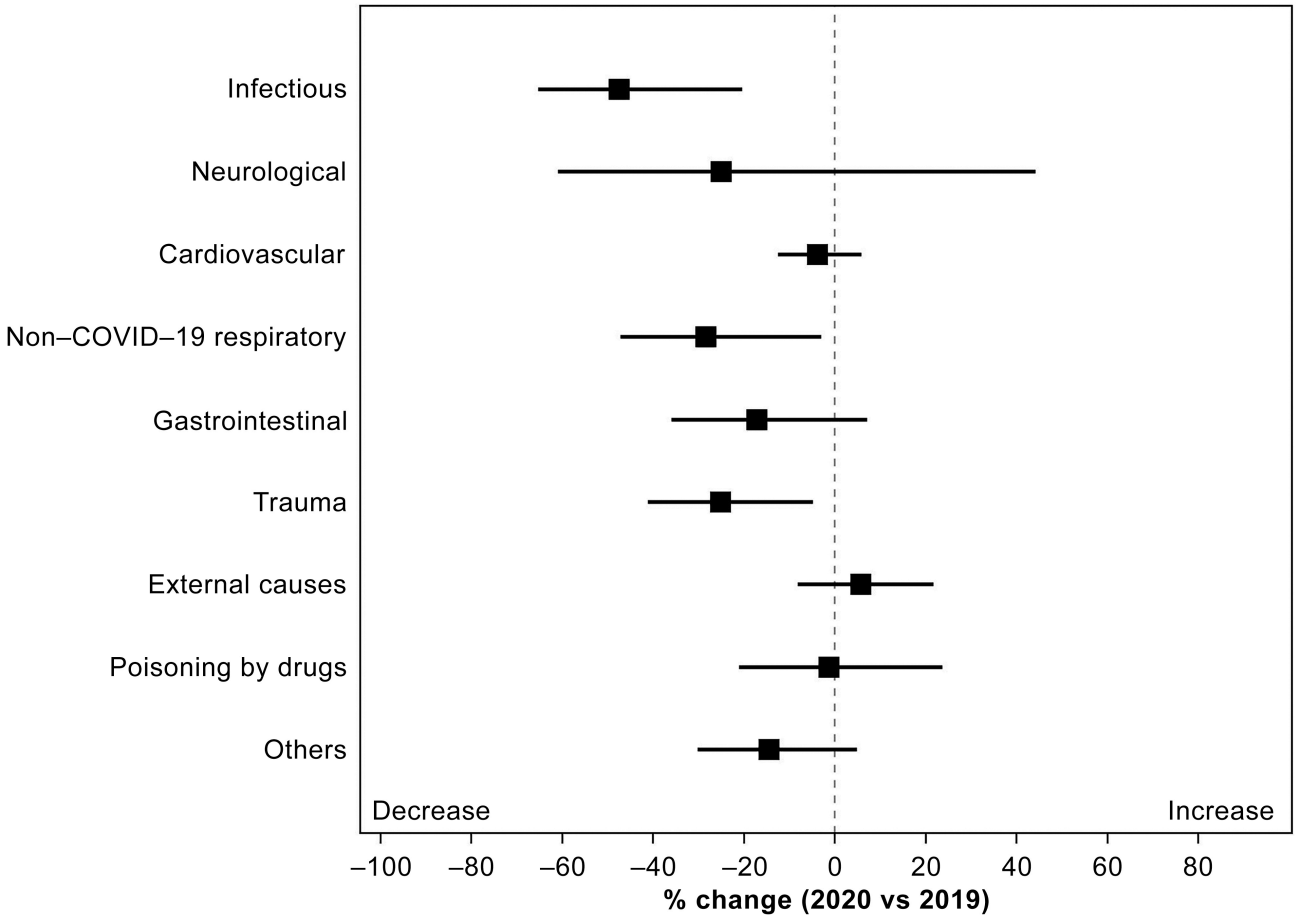
Percent change (with 95% confidence interval) in emergency department (ED) diagnoses other than COVID-19.

The patients transported to the tertiary ED were confirmed by diagnostic classification, and all diagnoses except External causes decreased in 2020 (Figure 4). Significant decreases were observed in Infectious (47.5%, p = 0.002), Non-COVID-19 respiratory (28.4%, p = 0.030), and Trauma (25.2%, p = 0.017). External causes increased (5.7%, p = 0.441) compared with that in the previous year.

### Changes in the number of suicides

Among External causes, only the rate of change in suicide cases increased by 43.2% (p = 0.089). Regarding the trend in suicide cases, compared with before the transmission of COVID-19, the ratio of women (32.4% vs. 47.2%; p = 0.162) increased, and the mean age showed a tendency to decrease (50.4 years vs. 48.7 years; p = 0.697), but no significant difference was observed in both (Table 3).

**Table 3.** Trends in Patients with Cardiopulmonary Arrest.

ED final diagnosis	ED visit count in 2019	ED visit count in 2020	% change (95% CI)	95% confidence interval		P-value
				Lower	Upper	
Total patient	2,184	2,013	−7.8%	−13.5	−1.7	0.013
External causes	368	389	5.7%	−8.2	21.7	0.441
All cardiopulmonary arrest cases	541	559	3.3%	−9.1	17.5	0.617
Cardiopulmonary arrest due to suicide	37	53	43.2%	−5.7	117.5	0.089

※Excluding data as of February 29, 2020

## Discussion

In this study, we evaluated the changes in the number of patients transported to an emergency and critical care center before and after the COVID-19 pandemic in Japan. Throughout the year, the number of patients transported to the tertiary ED reduced as a result of the COVID-19 pandemic. This trend was the same as that in Italy ^(7)^ and the United States ^(8), (9)^, where the number of confirmed cases was higher than that recorded in Japan.

First, we verified the number of patients transported to the tertiary ED by comparing it with the number of confirmed COVID-19 cases. The first wave of COVID-19 hit Japan in March-April 2020, and the number of patients transported to the tertiary ED in the same period decreased compared with that in the previous year. Subsequently, the number of patients transported to the tertiary ED in May, when the number of confirmed cases decreased, increased compared with that in the previous year. The same trend was observed from July to August 2020, when the second wave hit. Thus, the transition of the number of patients transported to the tertiary ED associated with COVID-19 was characterized by the fact that although it temporarily decreased during the period when the number of confirmed cases increased, a subsequent increase was observed. When examining the medical system of emergency and critical care centers, this suggests that the behavioral restriction brought about by COVID-19 affects the patient’s consultation behavior. Similar trends can be observed in studies from other countries. A study conducted in a Finnish ED reported a 16% reduction in post-lockdown emergency visits ^(17)^. According to a study conducted in a French ED, the number of patients transported with acute myocardial infarction and stroke decreased by approximately 20 ^(18)^. Of note, the number of emergency transport cases in Europe and Japan, where the number of people infected with COVID-19 was much higher ^(19)^, was the same. Thus, the change in the number of emergency transport cases owing to COVID-19 is not the result of the infectious disease itself, but the result of changes in the behavior of patients who are afraid of contracting the infection. Changes in consultation behavior in tertiary emergency care requiring advanced medical care pose a risk of aggravation. In addition, it is necessary to fully consider the burden on medical institutions 1-2 months after the number of infected people peaks.

Next, we verified the patients transported to the tertiary ED by diagnostic classification. Nearly all diagnostic categories showed a decrease compared with that in the previous year. In particular, Infectious, Non-COVID-19 respiratory, and Trauma, which significantly decreased, showed trends similar to those in a previous study ^(7)^. Lockdowns associated with COVID-19 reportedly reduced the number of trauma patients in the ED in many countries ^(20), (21)^. Thus, measures against the transmission of COVID-19 infection may provide secondary benefits to emergency medical care. In contrast, the transmission of COVID-19 has led to a new challenge in emergency medical care, which is the increase in External causes. External causes are classified as “External causes of morbidity and mortality” in the ICD-10 diagnostic categories, and most of them were cases of traumatic cardiopulmonary arrest. The decrease in trauma because of restrictions on people leaving their homes has previously been discussed, but cases of traumatic cardiopulmonary arrest have increased. The breakdown of the cases of cardiopulmonary arrest in this study was examined, and it was found that suicide cases had increased by more than 40%. The impact of COVID-19 on mental health has been reported from multiple viewpoints ^(22), (23)^. Among them, a report associated with COVID-19 and suicide stated that there is no cause-and-effect relationship between COVID-19 and the increase in the number of suicides ^(24)^. In the present study, the number of suicides also increased sharply, but the cause could be identified. The percentage of suicidal ideations after the transmission of COVID-19 infection is reportedly increasing ^(25)^, and the direct cause-and-effect relationship with COVID-19 is unknown; however, it should be monitored closely as a factor causing changes in emergency medical care in the future.

This study was a single-center retrospective observational study; thus, changes in the diagnostic classification could not be verified given the small sample size. Further, the numbers of ED visits in 2020 were compared only to those in 2019 as no data from 2018 or before were available. Thus, trends over time could not be evaluated. Because the COVID-19 situation may cause differences in the number of transported cases by diagnostic classification, large-scale studies are warranted in the future.

## Article Information

### Conflicts of Interest

None

### Sources of Funding

This study was supported by Research Grant for Healthcare Professional from the Kitasato University Hospital and Kitasato University East Hospital, Grant/Award Number: 2021-6

### Acknowledgement

We would like to express our gratitude to all the patients who cooperated in providing the data used in the present study. We also thank Crimson Interactive Pvt Ltd (Ulatus) for their assistance in manuscript translation and editing.

### Author Contributions

All authors contributed to the study conception and design. M. T. is the corresponding author and performed the data collection, made substantial contributions to the analysis and interpretation of the data, and wrote this manuscript. Y. H. performed the data collection and made contributions to the interpretation of the data. A. U. made substantial contributions to the analysis and interpretation of the data. T. M. performed the data collection and made contributions to the interpretation of the data. R. I. supported the analysis of the data and supervised the manuscript preparation. R. T. made contributions to the interpretation of the data. S. T. supported the analysis of the data and supervised the manuscript preparation. All authors read and approved the final manuscript.

### Approval by Institutional Review Board (IRB)

Institutional Review Board for Observation and Epidemiological Study, Kitasato University Medical Ethics Organization (B20-326).
